# Functional fitness and psychological well-being in older adults

**DOI:** 10.1186/s12877-024-05654-2

**Published:** 2025-01-04

**Authors:** Eliza E. Tóth, Aleksandar Vujić, Ferenc Ihász, Roberto Ruíz-Barquín, Attila Szabo

**Affiliations:** 1 Doctoral School of Psychology, ELTE Eötvös Loránd University Budapest, Budapest, Hungary; 2 Institute of Sport Science, Faculty of Education and Psychology, ELTE Eötvös Loránd University, Szombathely, Hungary; 3 Facultad de Formación de Profesorado y Educación, Universidad Autónoma de Madrid, Madrid, Spain; 4 Institute of Health Promotion and Sport Sciences, Faculty of Education and Psychology, ELTE Eötvös Loránd University, Bogdánfy St. 12, Budapest, H-1117 Hungary

**Keywords:** Ageing, Exercise, Fitness, Functionality, Mental health

## Abstract

**Background:**

Physical fitness and functioning are related to better mental health in older age. However, which fitness components (body composition, strength, flexibility, coordination, and endurance) are more closely related to psychological well-being (PWB) is unclear.

**Methods:**

This research examined how body mass index (BMI) and six indices of functional fitness (i.e., lower and upper body *strength*, lower and upper body *flexibility*, *coordination* [based on agility and balance], and aerobic *endurance*) relate to five psychological measures that could mirror PWB (i.e., resilience, mental well-being, optimism, life satisfaction, and happiness). Thirty-nine older adults (60–94 years; two-thirds female) were examined with the Fullerton Functional Fitness Test (FFFT) after completing five psychometric instruments.

**Results:**

Data were analyzed with correlations, ordinary least squares regressions, and regularized (elastic net) regressions, calculating the Lindeman, Merenda, and Gold (LMG) indices of the relative importance of the six FFFT components separately for the five psychological measures. Results revealed that BMI, upper body strength, and upper body flexibility were the least significant predictors of PWB. In contrast, endurance, complex movement coordination, and lower body flexibility emerged as the most significant predictors. Still, lower body strength correlated moderately positively with all PWB indices, and similarly, upper body flexibility with resilience, mental well-being, and happiness.

**Conclusions:**

These findings should stimulate research on the mechanism connecting functional fitness with PWB in older adults. Further, apart from their novelty, the findings could be valuable in providing directions for physical fitness intervention programs targeting mental and physical health for older people.

## Introduction

Caspersen et al. [1] first defined *physical fitness* as enjoying leisure activities, completing everyday tasks, and facing unexpected situations with sufficient energy without premature fatigue. Physical fitness is positively associated with mental health [2]. This relationship has also been demonstrated in older adults [3]. Physical fitness in older adults is maintained through regular physical activity [4], which is related to emotional resilience [5, 6] mental well-being (MWB) [5, 7, 8], optimism [8, 9], satisfaction with life [8, 10, 11], and happiness [10, 12].

However, most studies assessed only a few psychological indices and measured physical activity with questionnaires instead of objective functional fitness assessments. This approach is not optimal for establishing a robust connection between psychological well-being (PWB), physical fitness, and its components in older age. Physical fitness is generally mirrored via body composition, such as body mass index (BMI), strength, flexibility, coordination (agility, balance), and endurance [13], which are the core elements of *functional fitness* considered to be the most critical measure in exercise gerontology [14]. The Fullerton Functional Fitness Test (FFFT), also known as the ‘The Senior Fitness Test’ [15–17] measures functional fitness in older adults.

Healthcare professionals and fitness practitioners use the FFFT to identify areas that require improvement and create customized exercise programs that cater to the person’s unique needs. However, while the relationship between physical fitness and psychological health has received extensive attention in the literature [3], few studies have examined the connection between the six *components of functional fitness* and PWB. This association may be essential in designing physical exercise training programs that are also beneficial for preserving and promoting PWB in older adults.

Recent evidence shows that various *components* of physical fitness have a different relationship with psychological measures. For example, a study of older adults across multiple age groups revealed that three weekly participation in a fall-proof program lasting for eight weeks was associated with better *coordination* and MWB [18]. In addition, a recent study reported a positive relationship between satisfaction with life (SWL) and *coordination and endurance* based on the 2-minute step test [19]. Understanding the relative importance of functional fitness components in PWB can help inform physical activity intervention programs. However, such programs may also need to account for baseline levels of functional fitness and mental health, especially when individuals start from below-average physical condition and mental well-being and, hence, are more likely to experience improvements. This field study aimed to advance this understanding by examining associations between BMI, functional fitness components (measured objectively through FFFT), and PWB at a single time point, acknowledging that future longitudinal research could reveal additional insights regarding changes in mental health over time and across different baseline characteristics.

Although exploratory with an applied perspective, this research is based on the Cognitive Behavior Theory (CBT) [20], proposing that thoughts, emotions, behavior, and body sensations are critical factors in PWB. For example, better physical functionality is paired with more positive thoughts and emotions. In this regard, while flexibility and strengths are critically important, mobility, coordination, and endurance could be even more critical in self-assessment/appraisal of functional fitness and associated thoughts and emotions. Thus, we expected that FFFT components affect PWB measures differently. However, we posed no specific hypothesis about which component of functional fitness is most influential in each of the assessed PWB indices.

## Methods

### Participants

We sought permission from the management of five nursing homes randomly selected.^1^ on Google Maps within Fejér county in Hungary to conduct the study with their inhabitants who are fit for the research and wish to volunteer. Within two weeks, we received a response from two, one in Aba and one in Székesfehérvár. After obtaining permission, we visited the nursing home and verbally presented the research and the tasks participants must complete. Volunteers signed an informed consent form and a General Data Protection Regulation (GDPR) data handling form. In total, 39 older adults have completed the study. They were all 60 or over (*M*_*age*_ = 80.15 years; ± *SD* = 7.21 years, range 60 to 94 years), and two-thirds (*n* = 26) were women. Their mean weight was 78.00 ± 12.87 kg, height 1.70 ± 0.12 m, and BMI 27.11 ± 3.38. The institution’s doctor or head nurse screened the volunteers for health, considering the mental and physical conditions of the participants.

The physical criteria for exclusion were the inability to stand or walk, upper or lower limb deficits, medical conditions predisposing the person to dizziness, cardiac risk factors, loss of balance, and untreated hypertension. The psychological criteria for exclusion consisted of previously diagnosed mental and behavioral disorders. All participants had medical clearance for the study. Table 1 presents their demographic characteristics.

**Table 1:** Demographic characteristics of the participants and sitting/walking habits during the week preceding the study

Measures	Categories	Men (*n* = 13, 33.3%)	Women (*n* = 26, 66.7%)	Total
Education	Elementary school	1 (7.7%)	15 (57.7%)	16 (41.0%)
	High school	11 (84.6%)	11 (42.3%)	22 (56.4%)
	University	1 (7.7%)	0	1 (2.6%)
Civil status	Single	2 (15.4%)	2 (7.7%)	4 (10.3%)
	Married	1 (7.7%)	0	1 (2.6%)
	Divorced	3 (23.1%)	0	3 (7.7%)
	Widowed	6 (46.2%)	23 (88.5%)	29 (74.4%)
	Living with a partner	1 (7.7%)	1 (3.85%)	2 (5.1%)
Perceived health	Very good	1 (7.7%)	0	1 (2.6%)
	Good	6 (46.2%)	6 (23.1%)	12 (30.8%)
	Satisfactory	4 (30.8%)	17 (65.4%)	21 (53.8%)
	Bad	2 (15.4%)	2 (7.7%)	4 (10.3%)
	Very bad	0	1 (3.85%)	1 (2.6%)
Sitting (hours/day; estimate) *	1–2 h	0	0	0
	3–4 h	2 (15.4%)	0	2 (5.1%)
	5–6 h	6 (46.2%)	13 (50%)	19 (48.7%)
	More than 6 h	5 (38.5%)	13 (50%)	18 (46.2%)
Walking at least 10 min/day *	1–2 days	0	0	0
	3–4 days	1 (7.7%)	1 (3.85%)	2 (5.1%)
	5–6 days	0	4 (15.4%)	4 (10.3%)
	Every day, at least once	12 (92.3%)	21 (80.8%)	33 (84.6%)
	Did not walk at all	0	0	0
Estimated total daily walk *	Less than one hour	5 (38.5%)	13 (50%)	18 (46.2%)
	1–2 h	7 (53.8%)	13 (50%)	20 (51.3%)
	3–4 h	1 (7.7%)	0	1 (2.6%)
	More than 4 h	0	0	0
	Did not walk	0	0	0
		**Men** **(Mean ± SD)**	**Women (Mean ± SD)**	**Total** **(Mean ± SD)**
Lower body strength (FL1)		14.69 ± 5.01	10.69 ± 3.98	12.03 ± 4.68
Upper body strength (FL2)		15.54 ± 3.28	16.17 ± 3.94	15.96 ± 3.70
Upper body flexibility (FL3)		-16.62 ± 9.35	-22.85 ± 9.37	-20.77 ± 9.68
Lower body flexibility (FL4)		-2.31 ± 2.77	-4.34 ± 5.07	-3.67 ± 4.50
Complex coordination (FL5)		11.77 ± 4.71	16.12 ± 5.27	14.67 ± 5.44
Endurance (FL6)		89.38 ± 29.01	73.15 ± 21.51	78.56 ± 25.11

Note * These questions mirror the subjective estimate for the week preceding the study. SD = standard deviation

#### Ethics

The study was conducted in December 2022 with approval (permission No. 2022/510) from the Faculty of Education and Psychology Research Ethics Board at Eötvös Loránd University. The work conformed to the ethical guidelines of the British Psychological Society (BPS) Code of Human Research Ethics [21]. Additionally, the protocol followed the research principles with human participants of the Helsinki Declaration [22].

#### Measures

The dependent measures comprised *subjective* responses collected with questionnaires and *objective* functional fitness assessments.

#### Subjective measures

Apart from demographic questions (see Table 1), mental well-being (MWB) was measured using the Mental Health Continuum-Short Form [23, 24]. The 25-item Connor-Davidson Resilience Scale [25, 26] assessed resilience. Optimism was gauged with the Life Orientation Test (LOT-R) [27, 28], while SWL was measured with the Satisfaction with Life Scale [29, 30]. Finally, the perceived happiness was estimated with the Subjective Happiness Scale [31, 32]. All instruments have good psychometric properties (see their sources) that are not described here for parsimony.

#### Objective measures

The FFFT evaluates older adults’ functional fitness by assessing upper and lower body strength, upper and lower body flexibility, complex coordination, and endurance. The test consists of tasks, such as walking, lifting weights, reaching, and balancing, which are essential in daily life activities. Research evidence based on measures of stability, reliability, and discriminant validity supports the validity of the FFFT [14]. In this study, we used the FFFT to assess functional fitness [15, 16, 33] through six measures:

1. *Lower body strength****(FL1)***: 30s chair test, complete stand up and sit down (number of repetitions).
2. *Upper body strength****(FL2)***: lifting 2 (women) or 3.5 (men) kg dumbbell while sitting on a chair and doing complete arm bends and stretches (number of repetitions in 30 Sect. ).
3. *Upper body flexibility****(FL3)***: fingers touching behind the back (back scratch) (+/- cm).
4. *Lower body flexibility****(FL4)***: forward bend from chair to extended leg (chair sit-and-reach) (+/- cm).
5. *Complex coordination (agility*,* balance*,* and walking speed)****(FL5)*** – circling a cone, which is located 2.44 m (8 feet) from the starting position, in the shortest possible time and then returns to the starting position.
6. *Endurance (physical effort****(FL6)***: 2-minute step test - records the number of whole steps completed in two minutes, raising each knee to the point halfway between the patella (knee-cap) and iliac crest (top hip bone).

The FFFT is safe for both inactive and physically active older adults. Moreover, by using everyday motor patterns, researchers can get an insight into the six functional fitness indices described above [34]. Finally, we calculated BMI by dividing participants’ weight (kg) by height (m) squared.

### Procedure

Data collection occurred individually in a quiet room in the participants’ habitual environment. While completing the questionnaires, a researcher was present, but she did not interact with the participants unless they had questions about their tasks. After the participants completed the questionnaires presented in shuffled (random) order and answered the demographic questions, the researcher explained the FFFT before each of the six trials. Next, she demonstrated the correct execution of the upcoming task. Each FFFT trial was performed twice and the better performance on the two trials was recorded. After completing twice each of the six FFFT trials, the participants were debriefed and thanked for participating.

### Data analyses

After testing the normal distribution of the results, we examined possible gender differences in the dependent measures using the Mann-Whitney U tests. Subsequently, we calculated Pearson’s correlations between the observed variables. Regression analyses were conducted in R programming language [35], using ‘tidyverse’ [36], ‘glment‘ [37], ‘glmnetUtils’ [38], and ‘relaimpo‘ [39] packages. Five regressions examined the relative importance of FFFT and BMI scores in predicting five psychological measures. Since there was a strong multicollinearity present in each model, it was impossible to distinguish between each predictor’s importance by looking at their coefficients and *p*-values. For example, the *R*^*2*^ was high in all instances, but the individual predictors were insignificant.

Therefore, to unveil the relative importance of each FFFT component and BMI, we ran a series of regularized regressions (elastic net) in addition to ordinary least squares (OLS) regressions [40]. In an elastic net (the combination of ridge and lasso regressions), multicollinearity usually does not present a problem. We can see the predictors’ importance by combining this method with the LMG relative importance metric (see [41]). Although coefficients from regularized regression cannot be easily interpreted as OLS coefficients, they can help identify *essential contributors* and the *direction* in which they relate to the outcome measures. Five-fold cross-validation was used to determine optimal values for the hyperparameters alpha and lambda (see [40]). However, for SWL and happiness, the alpha was increased from 0.0 to 0.1 to avoid fitting a complete ridge regression model.

The LMG metric is independent of the order of predictors in the model. It can represent a relative proportion (summing to 1) or relate to the model’s *R²*, with LMG values adding up to the model’s *R²*. In contrast to regression coefficients, which can be distorted and yield reversed signs in multicollinearity, the importance measures derived from LMG are always positive and provide a more suitable decomposition of the model’s *R²* than standardized regression coefficients. Further, multicollinearity can inflate *p*-values, indicating nonsignificant predictors, while the overall model’s *F*-test remains significant [41, 42]. Therefore, we utilized regularized regression models, such as elastic net, to effectively identify the most influential predictors in multicollinear contexts, supplementing our analysis and avoiding misleading results from ordinary least squares (OLS).

## Results

### Normality test

Based on Kolmogorov-Smirnov normality tests with Lilliefors significance correction, the normal distribution assumption was violated only in lower body flexibility (FL4, *p* < .001) and endurance (FL6, *p* = .04) but no other FFFT or psychological measures. However, skewness (FL4 = -1.05 and FL6 = 0.637) and kurtosis (FL4 = -0.096 and FL6 = -0.441) values suggested negligible violation of the normality assumption in these measures and that practically the data can be considered relatively normally distributed even in these two measures.

### Gender differences

Due to the small sample size, we opted for nonparametric tests to examine possible gender differences in the outcome measures. None of the Bonferroni-corrected Mann-Whitney U tests was statistically significant. Hence, we did not pursue further gender differences and performed the subsequent statistical tests for the whole sample.

### Correlations

Pearson’s correlations indicated that the psychological measures were positively correlated, ranging from *r* = 0.48 to 0.78. The BMI did not correlate significantly with any FFFT or PWB measures. Furthermore, the correlations between FFFT components were positive, and apart from one (FL4 and FL2), all were statistically significant (Table 2). Several PWB measures correlated statistically significantly with FFFT measures. In the case of FL5, the negative correlations indicate a positive connection (shorter time means better coordination).

**Table 2:** Correlations between physical functionality tests, BMI, and psychological measures

	Pearson’s *r* correlation	*p* (2-tailed)	95% Confidence Intervals ^a^	
			Lower	Upper
FL1 - FL2	0.594	< 0.001	0.342	0.766
FL1 - FL3	0.458	0.003	0.166	0.676
FL1 - FL4	0.365	0.022	0.056	0.610
FL1 - FL5	-0.703	< 0.001	-0.834	-0.498
FL1 - FL6	0.660	< 0.001	0.435	0.807
FL1 - BMI	-0.280	0.085	-0.547	0.039
FL1 - Resilience	0.507	0.001	0.228	0.709
FL1 – Mental well-being	0.452	0.004	0.160	0.672
FL1 - Optimism	0.275	0.090	-0.044	0.543
FL1 – Satisfaction with life	0.520	0.001	0.245	0.718
FL1 - Happiness	0.411	0.009	0.109	0.643
FL2 - FL3	0.430	0.006	0.132	0.656
FL2 - FL4	0.147	0.373	-0.177	0.442
FL2 - FL5	-0.484	0.002	-0.693	-0.198
FL2 - FL6	0.466	0.003	0.176	0.681
FL2 - BMI	-0.177	0.282	-0.466	0.147
FL2 - Resilience	0.281	0.083	-0.038	0.548
FL2 - Mental well-being	0.239	0.142	-0.082	0.516
FL2 - Optimism	0.059	0.721	-0.261	0.368
FL2 - Satisfaction with life	0.344	0.032	0.032	0.595
FL2 - Happiness	0.197	0.230	-0.126	0.482
FL3 - FL4	0.512	0.001	0.234	0.712
FL3 - FL5	-0.705	< 0.001	-0.835	-0.502
FL3 - FL6	0.516	0.001	0.240	0.715
FL3 - BMI	-0.173	0.292	-0.463	0.151
FL3 - Resilience	0.355	0.027	0.044	0.603
FL3 - Mental well-being	0.410	0.010	0.109	0.643
FL3 - Optimism	0.174	0.289	-0.150	0.464
FL3 - Satisfaction with life	0.279	0.085	-0.040	0.547
FL3 - Happiness	0.357	0.026	0.047	0.605
FL4 - FL5	-0.628	< 0.001	-0.787	-0.389
FL4 - FL6	0.529	0.001	0.256	0.723
FL4 - BMI	-0.179	0.275	-0.468	0.145
FL4 - Resilience	0.476	0.002	0.189	0.688
FL4 - Mental well-being	0.507	0.001	0.228	0.709
FL4 - Optimism	0.369	0.021	0.060	0.613
FL4 - Satisfaction with life	0.465	0.003	0.176	0.681
FL4 - Happiness	0.504	0.001	0.224	0.707
FL5 - FL6	-0.824	< 0.001	-0.904	-0.686
FL5 - BMI	0.244	0.135	-0.078	0.519
FL5 - Resilience	-0.606	< 0.001	-0.774	-0.359
FL5 - Mental well-being	-0.512	0.001	-0.713	-0.234
FL5 - Optimism	-0.307	0.057	-0.568	0.009
FL5 -Satisfaction with life	-0.564	< 0.001	-0.747	-0.303
FL5 - Happiness	-0.516	0.001	-0.715	-0.240
FL6 - BMI	-0.291	0.072	-0.555	0.027
FL6 - Resilience	0.642	< 0.001	0.409	0.796
FL6 - Mental well-being	0.532	< 0.001	0.260	0.725
FL6 - Optimism	0.499	0.001	0.217	0.703
FL6 - Satisfaction with life	0.681	< 0.001	0.466	0.820
FL6 - Happiness	0.524	0.001	0.250	0.721
BMI - Resilience	-0.165	0.316	-0.457	0.159
BMI - Mental well-being	-0.077	0.643	-0.383	0.245
BMI - Optimism	-0.013	0.939	-0.327	0.304
BMI - Satisfaction with life	-0.270	0.096	-0.540	0.050
BMI - Happiness	-0.218	0.183	-0.499	0.105
Resilience – Mental well-being	0.780	< 0.001	0.616	0.879
Resilience - Optimism	0.605	< 0.001	0.357	0.773
Resilience - Satisfaction with life	0.599	< 0.001	0.350	0.769
Resilience - Happiness	0.608	< 0.001	0.362	0.775
Mental well-being - Optimism	0.562	< 0.001	0.299	0.745
Mental well-being - Satisfaction with life	0.592	< 0.001	0.341	0.765
Mental well-being- Happiness	0.614	< 0.001	0.371	0.779
Optimism - Satisfaction with life	0.489	0.002	0.205	0.697
Optimism - Happiness	0.575	< 0.001	0.317	0.754
Satisfaction with life - Happiness	0.769	< 0.001	0.598	0.873

Note ^a^ Estimation is based on Fisher’s r-to-z transformation; BMI = Body mass index; FL1 = Lower body strength; FL2 = Upper body strength; FL3 = Upper body flexibility; FL4 = Lower body flexibility; FL5 = Complex coordination; FL6 = Endurance

### Ordinary least squares (OLS) regressions

As shown in Table 3, only FL6 was significant in only two models despite all predictors having a high correlation with PWB variables. So, due to multicollinearity, we cannot determine the importance of the predictors based on OLS regression results.

**Table 3:** Ordinary least squares regressions – multicollinearity present (*n* = 39)

Predictors	Resilience		Mental well-being		Optimism		Life satisfaction		Happiness	
	b	*p*	b	*p*	b	*p*	b	*p*	b	*p*
Intercept	1.93	0.070	3.41	**0.011**	0.95	0.439	2.37	0.270	4.43	0.056
FL1	0.02	0.484	0.03	0.287	0.02	0.508	0.03	0.525	0.03	0.594
FL2	-0.01	0.766	-0.01	0.728	-0.03	0.350	0.02	0.691	-0.02	0.674
FL3	-0.00	0.629	0.01	0.537	0.00	0.895	-0.02	0.297	0.00	0.981
FL4	0.02	0.351	0.04	0.118	0.03	0.200	0.06	0.189	0.06	0.153
FL5	-0.02	0.604	0.02	0.635	0.05	0.144	0.01	0.902	-0.01	0.881
FL6	0.01	0.110	0.01	0.197	0.02	0.005	0.02	0.021	0.01	0.320
BMI	0.01	0.783	0.02	0.437	0.03	0.307	-0.02	0.639	-0.02	0.720
*R*^*2*^ / *R*^*2*^ adjusted	0.460 / 0.338		0.391 / 0.253		0.369 / 0.227		0.517 / 0.408		0.359 / 0.215	

Note FL1 to FL6 = Fullerton test forms; BMI = Body mass index; *b* = unstandardized regression coefficient; *p* = *p-value*

### Elastic Net regressions and LMG relative importance

#### Resilience

**Fig. 1:**
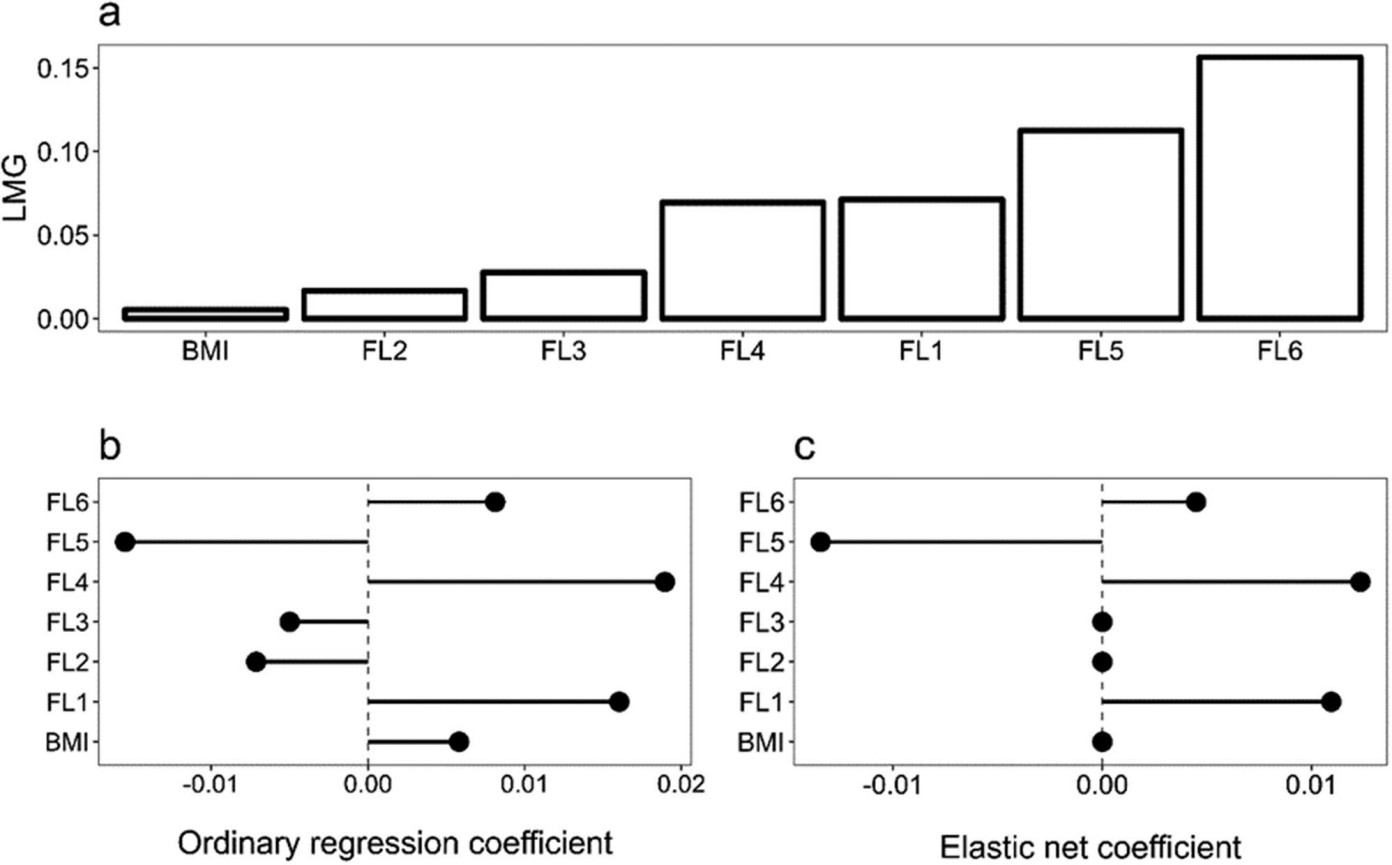
Resilience - LMG Relative Importance (**a**), OLS Coefficients (**b**), and Elastic Net Coefficients (**c**) for resilience

Concerning resilience, both the LMG method and elastic net model indicated that the FL6, FL5, FL1, and FL4 were the most important predictors (Fig. 1, a, c), while FL3, FL2, and BMI were the least important predictors. As such, FL3, FL2, and BMI were shrunk to zero in the elastic net model. FL5 negatively affected the outcome since a shorter time indicates better performance. Optimal values for lambda and alpha hyperparameters were 0.275 and 0.125, respectively (Fig. 1).

#### Mental well-being

**Fig. 2:**
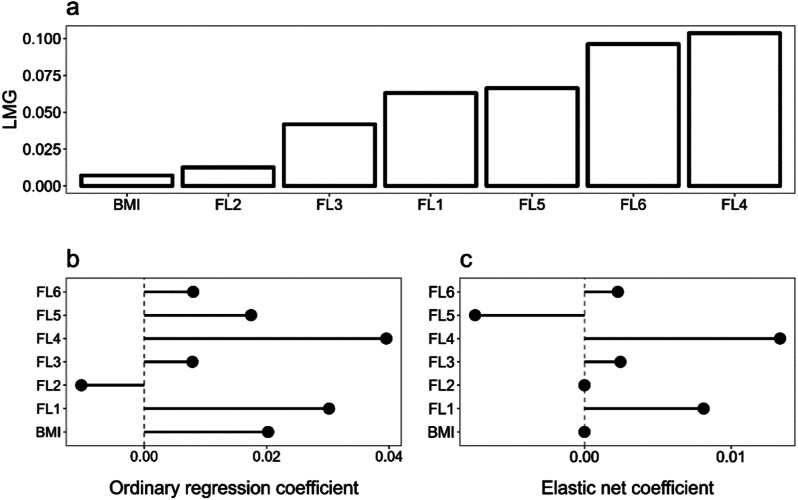
Mental well-being - LMG Relative Importance (**a**), OLS Coefficients (**b**), and Elastic Net Coefficients (**c**) for mental well-being

As for MWB, FL4 and FL6 appeared to be the most important, while again, FL2 and BMI were the least important predictors. FL3 was not shrunken to zero in the elastic net model, having roughly equal magnitude as FL6, although the LMG metric indicated FL6 had much greater importance than FL3. Finally, FL1 and FL5 could be equally important predictors. The best lambda value for this model was 0.789, and the alpha was set to 0.1 (Fig. 2).

#### Optimism

**Fig. 3:**
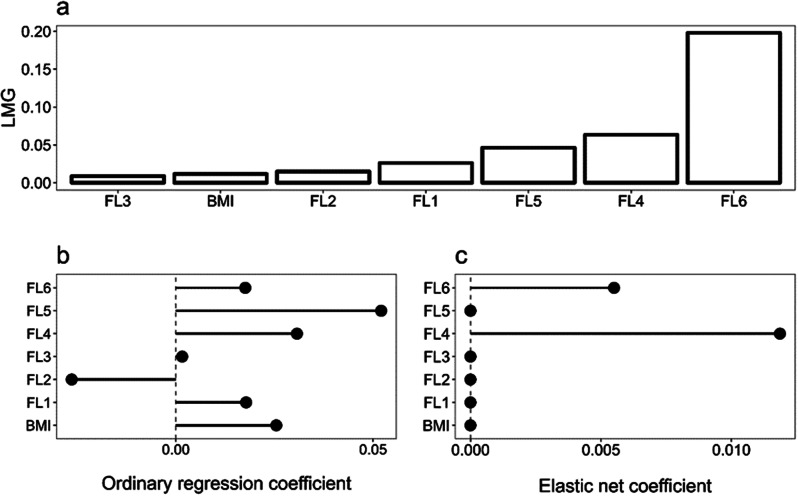
Optimism - LMG Relative Importance (**a**), OLS Coefficients (**b**), and Elastic Net Coefficients (**c**) for optimism

Regarding Optimism, FL6 was shown to be the most important predictor, and its contribution was notably larger than the rest, followed by FL4 and FL5. Again, FL2, FL3, and BMI were the least important. However, the elastic net shrunk all but FL6 and FL4 predictors to zero. Alpha and lambda hyperparameters for this model were 0.216 and 0.258 (Fig. 3).

#### Satisfaction with life (SWL)

**Fig. 4:**
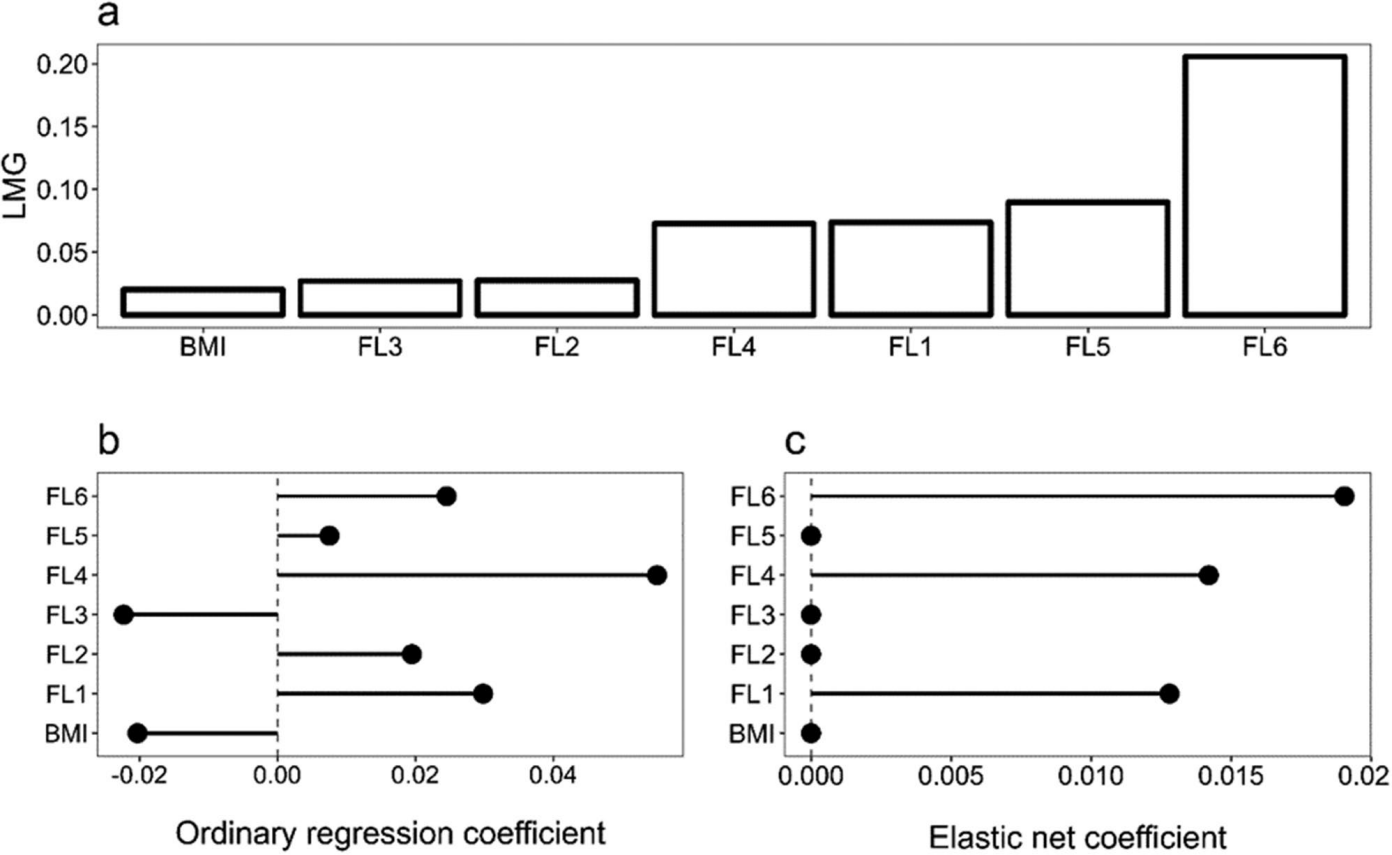
Satisfaction with life - LMG Relative Importance (**a**), OLS Coefficients (**b**), and Elastic Net Coefficients (**c**) for satisfaction with life

For SWL, FL6 was again the most important, followed by FL5 and FL1. BMI, FL2 and FL3 were, again, the least relevant predictors. Surprisingly, FL1 played a more important role than for the other well-being outcomes (with the exception of resilience), and FL5 was reduced to zero in the elastic net regression model. Optimal values for alpha and lambda were 0.729 and 0.228 (Fig. 4).

#### Happiness

**Fig. 5:**
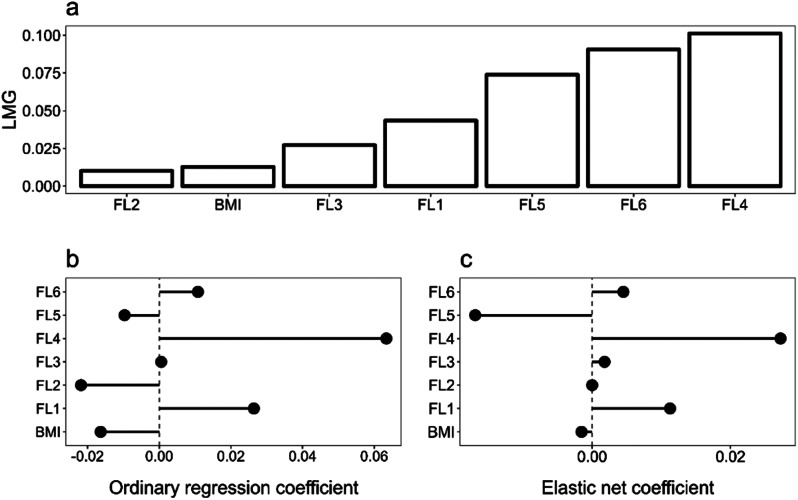
Happiness - LMG Relative Importance (**a**), OLS Coefficients (**b**), and Elastic Net Coefficients (**c**) for happiness

FL4 seemed the most important for Happiness, followed by FL6 and FL5. Once again, FL2, BMI, and FL3 appeared to be the least important in predicting this outcome. Only FL2 was shrunk to zero in the elastic net. As for happiness, the chosen alpha value was set to 0.1, and the optimal value for lambda was 1.223 (Fig. 5).

## Discussion

The current study examined BMI and specific components of functional fitness, including strength, flexibility, coordination, and endurance, and their relationship to five indices of PWB, including resilience, MWB, optimism, SWL, and happiness in older adults. The critical finding reflecting the unique contribution of this research is that endurance, complex coordination, and lower body flexibility surface as the most influential factors in PWB in older adults. These results agree with a recent report disclosing a significant relationship between SWL and body coordination and endurance [19]. However, the current study expands those results because it tested additional psychological measures from which a relatively consistent picture emerged concerning the relative importance of functional fitness components to PWB.

The correlation results indicated that psychological resilience had the most robust relationship with complex coordination (FL5) and endurance (FL6). The correlation coefficients and their 95% confidence intervals (CI) suggest a reliable, moderate to strong relationship between resilience and FL5 and resilience and FL6 (see Table 2). The inverse correlation with FL5 is because a shorter completion time of the FL5 task reflects a better performance. Furthermore, MWB, SWL, and happiness exhibited comparable statistically significant correlations with FL5 and FL6 and with upper and lower body flexibility (FL3 and FL4) and lower body strength (FL1). Based on the 95% CIs, the relationships are consistent in direction (i.e., positive) but can vary from weak to strong (refer to Table 2). Optimism was most closely associated with endurance (FL6) based on a statistically significant moderate positive correlation. Examining the 95% CI, these relationships appear to be directionally consistent and could range in strength from weak to moderately strong. Thus, the correlation results suggest a positive association between functional fitness and PWB, but the relationship requires further research with larger samples.

Our results, based on correlations and regularized elastic net regressions, suggest that BMI, upper body strength, and upper body flexibility may be the least essential predictors of PWB. Endurance, however, was always among the top two most important predictors based on LMG. Furthermore, it was the critical predictor of optimism and SWL. However, lower body strength and flexibility are also associated with these measures. Furthermore, lower body strength correlated statistically significantly with all PWB measures, but it only emerged as a weak or moderate predictor in the regression models. Additionally, lower body flexibility was the primary predictor of MWB and happiness. Notable is that, in SWL and optimism, endurance had a remarkably higher LMG value than the rest. This trend was not the case with the other outcomes, where the highest importance was relatively balanced with either coordination or lower body flexibility measures. These results indicate that the three most important predictors of PWB were endurance, complex movement coordination, and lower body flexibility, except for SWL and resilience, for which endurance, coordination, and lower body strength were the top three. Finally, BMI, upper body strength, and upper body flexibility had the lowest importance values in the battery of PWB measures.

Since no similar studies have been conducted before, except for Gacek et al. [19], the results cannot be compared to past research. However, they indicate that BMI is *not* an essential factor in the PWB of older adults. Instead, *endurance* (aerobic), *complex movement coordination* (agility and balance), and lower body flexibility appear to be instrumental in multiple measures of PWB. Lower body flexibility was the most critical predictor of four out of five PWB measures *based on elastic net regression*, and endurance was the chief predictor *based on LMG*. Future studies should replicate the current one with larger samples because the mechanism connecting lower body flexibility with PWB is unknown, and, therefore, it should be identified. On the other hand, the role of endurance is understandable and expected based on CBT. The current results could also serve as a direction for future physical intervention programs for older adults. They should target endurance, complex coordination, and lower body flexibility since their improvement might benefit PWB the most. However, this contention requires longitudinal intervention research, and more PSW indices should be assessed.

### Limitations

The current research has potential limitations that should be considered when interpreting the results. First, the findings stem from volunteers in two nursing homes who may not represent the target population. Second, the sample is relatively small, which makes the regularized regression results less stable, requiring caution when interpreting the coefficients. Third, considering regularized models, ridge regression tries to shrink the collinear variables, while lasso regression tends to drive one or more coefficients of the collinear predictors to zero [40]. A compensatory solution used here was that since elastic net regression combines these two approaches, selecting the optimal parameters finds the trade-off between sharing the credit among predictors and shrinking correlated variables to zero, but the coefficients have no straightforward interpretation [40]. Fourth, the standard endurance test [16] we used (i.e., FL6) may be more than just an endurance test as it could be a challenging dynamic balance task for some older adults.

## Conclusions

Different components of functional fitness in older adults relate differently to measures of PWB. It appears that BMI, upper body strength, and upper body flexibility are the least significant predictors of PWB. In contrast, endurance, complex coordination, and lower body flexibility emerge as the most important predictors of PWB measures. Future research examining larger and more representative samples should replicate the current finding to enable scholars and healthcare professionals to design the most optimal physical activity intervention program for older adults that improves their functional fitness and simultaneously fosters the enhancement of their PWB.

## Data Availability

The data have been deposited to the Mendeley data repository (10.17632/kpx5pbwkvz.1).

## Footnotes

1 After Google Maps listed the establishments in the region, we drew five blinded numbers (each folded in a piece of paper in a glass jar) from among the total listed (34).
